# Validation of the use of p-aminobenzoic acid to determine completeness of 24 h urine collections in surveys of diet and nutrition

**DOI:** 10.1038/s41430-018-0195-x

**Published:** 2018-06-05

**Authors:** Lorna Cox, Kate Guberg, Stephen Young, Sonja Nicholson, Toni Steer, Ann Prentice, Polly Page

**Affiliations:** Medical Research Council Elsie Widdowson Laboratory, 120 Fulbourn Road, Cambridge, CB1 9NL UK

**Keywords:** Biomarkers, Epidemiology

## Abstract

Sodium intake is assessed using 24 h urinary excretion; it is important to ensure urine collections are complete. This can be validated by monitoring urinary excretion of p-aminobenzoic acid (PABA) administered in tablet form at intervals during the urine collection. Unavoidable change of PABA tablet supplier and analytical procedure required re-establishment of the thresholds consistent with a complete collection. Reference ranges for adults without reported intestinal or renal disease were determined by HPLC (70–103%) and colorimetry (84–120%). Some individuals excreted a small, measurable amount of PABA the following day but this did not represent the balance of the PABA ingested. Assay of the PABA tablets confirmed the stated dose (80 mg) and demonstrated their stability up to 8 years (duration of study) at room temperature. These tablets have been used and the reference ranges applied in UK national population surveys since 2008.

## Introduction

Assessment of dietary intake, such as sodium, by measuring 24 h urinary excretion depends on identifying those 24 h collections which are complete. Urinary excretion of administered p-aminobenzoic acid (PABA) has been used as a marker which can distinguish between complete and incomplete 24 h urine collections [1–5] and as a reference against which the performance of other candidate criteria has been judged [6]. The original assay method for PABA analysis was based on colorimetry; HPLC was later introduced [7] to improve specificity.

The purpose of this study was to establish appropriate criteria for the use of PABA tablets from a new supplier in the UK National Diet and Nutrition Survey Rolling Programme (NDNS RP, 2008–2013) (https://www.gov.uk/government/statistics/national-diet-and-nutrition-survey-assessment-of-dietary-sodium-in-adults-in-england-2014; https://www.gov.uk/government/statistics/national-diet-and-nutrition-survey-results-from-years-1-to-4-combined-of-the-rolling-programme-for-2008-and-2009-to-2011-and-2012), for which both adults and children were asked to provide complete 24 h urine collections for measurement of sodium, potassium and nitrogen excretion and estimation of daily salt intake.

In addition, collection of urine for a second 24 h period the following day was undertaken, to explore the fate of ingested PABA not excreted in the urine the same day.

## Subjects, materials and methods

51 volunteers (19 M, 32 F) aged 19–64 years were recruited (conventionally regarded as sufficient to define mean and SD of a Normal distribution), excluding individuals who had intestinal or renal disease or were pregnant. Each gave fully informed written consent. Ethical approval was obtained from the Cambridge Local Research Ethics Committee. Each was instructed to take three 80 mg PABA tablets during a single day, at breakfast time, lunchtime and at 6–8 p.m. respectively. They were also given detailed instructions regarding collecting 24 h urine on the day of PABA ingestion (“study day”) and on the day following (“next day”) and an explanation of the importance of providing a complete collection. Each subject recorded start and finish times for each day, time of taking PABA, details of other drug and multivitamin tablet ingestion and whether any urine had been missed from the collection.

### PABA tablets

Due to the previous established supplier having discontinued tablet production, 80 mg PABA tablets were purchased from Lonsdale Health Products Ltd (Units 4/5A/5B, Ingleton Industrial Estate, LA6 3NU) and packaged into 3-tablet blister packs. Three manufacturing batches were examined. PABA content was measured in 10 tablets on each occasion, randomly selected on receipt and annually for 8 up to years; each tablet was dissolved in NaOH and assayed by HPLC. PABA content was confirmed at all time-points, within experimental error; there was no evidence of a decrease in the mean PABA content over 8 years at room temperature. (Data not shown). This was taken as evidence of the suitability of this brand of PABA tablets for this experiment and for use in NDNS and other surveys and studies.

### PABA: colorimetric assay method

The colorimetric assay for PABA was an in-house adaptation to microplate format of the method of Bingham and Cummings [1] using an iEMS plate—reader with 530 ± 5 nm filter. Other endogenous amines can interfere in this method, notably paracetamol and sulphonamides. Analytical imprecision as determined from single-use aliquots of urine containing excreted PABA, stored frozen and assayed in every analytical batch over a period of two years including this study, was 13.4% (cv) at 100 mg/l. The limit of quantitation of the assay was 20 mg/l.

### PABA: HPLC method

This assay is based upon the method described by Jakobsen [7] modified to replace the acetonitrile in the mobile phase with methanol. PABA metabolites in urine were hydrolysed under alkaline conditions; the solution was then neutralised and the resultant PABA determined by reverse-phase HPLC on a C18 column using meta-hydroxybenzoic acid as internal standard and mobile phase 70% phosphate buffer pH 3.5: 30% methanol. Quantitation was by absorbance at 290 nm. Completeness of hydrolysis was monitored by including a sample containing PAHA (para-aminohippuric acid) with each batch. This is hydrolysed to PABA which is then quantitated by HPLC. Analytical imprecision determined as above was 5.7% at 75 mg/l (*n* = 20), 8.7% at 30 mg/l (*n* = 15) and 19.5% at 14 mg/l (*n* = 15). The limit of quantitation was 6 mg/l.

PABA excretion was calculated by multiplying concentration by 24 h volume. The percentage of the PABA dose excreted was then calculated from urinary PABA content (100% = 240 mg).

## Results and discussion

49 urine collections made on the day PABA was taken were claimed by the participant to be complete collections. PABA content in these collections was shown to be Normally distributed.

HPLC reference range—Two collections with 24 h PABA more than 3 SD below the mean were excluded as outliers, as the most likely explanation was unrecorded “missed” urine. Inter-assay imprecision was added to the biological variation to determine overall SD. PABA excretions were normally distributed; the reference range was therefore defined as mean excretion ± two SD (*n* = 47).

Colorimetric reference range—Imprecision of the colorimetric assays in which these samples (*n* = 47) were assayed was similar to the expected between-batch imprecision of this assay and therefore the results obtained already included analytical imprecision. Two further collections, during which paracetamol had been taken, were excluded. The colorimetric reference range was therefore based upon *n* = 45 subjects. Use of a filter with a narrower bandwidth or of a diffraction-grating spectrophotometer would result in a more specific assay with a decreased upper limit to the reference range.

Table 1 shows the mean and SD PABA excretion and the reference ranges thereby derived for the HPLC and colorimetric assays. The lower limit for HPLC comprising 95% of complete collections (70% of nominally 240 mg dose) is lower than the 78% reported by Jakobsen et al. [7], reflecting our inclusion of analytical as well as biological imprecision in the estimate of SD.

**Table 1:** PABA recovery and reference range calculations

Assay method	**HPLC** ^a^	**Colorimetry** ^b^
*n*	47	45
Mean (% dose excreted)	86.5	102
SD (% dose excreted)	8.1	8.84
Mean–2SD (% dose excreted)	70	84
Mean − 3 SD (% dose excreted)	62	75
Mean + 2 SD (% dose excreted)	103	120
±2 SD range (% dose excreted)	70–103	84–120

^a^ Including between-batch analytical SD (6 mg/l) in total SD

^b^ Excluding two urines containing paracetamol

**Fig. 1:**
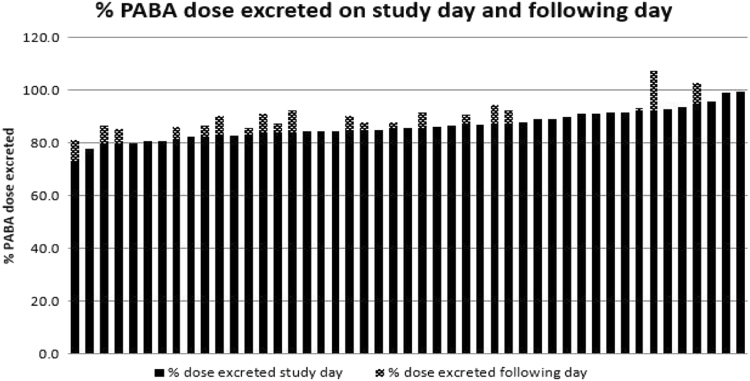
PABA excretion on the day following PABA ingestion

Delayed excretion—PABA was detectable by HPLC in 24 h urine collected the day after PABA ingestion by 20 of the 47 subjects whose collections on the study day were deemed complete, but this was not sufficient to account for the lower PABA excretion by some volunteers on the study day (Fig. 1). Therefore delayed urinary excretion was not the predominant cause of below-average PABA recovery in these volunteers, suggesting that lower PABA excretion within the reference range may result from decreased net absorption of PABA from the gut, possibly indicating bacterial metabolism of PABA.
